# PD204 Comparison Of Reimbursement Systems In France Denmark, Norway, And The United Kingdom - Possibilities Of Implementation In Poland

**DOI:** 10.1017/S0266462324004264

**Published:** 2025-01-07

**Authors:** Magdalena Rdzanek, Magdalena Chmielewska, Marcin Dawicki, Aleksandra Zawada

## Abstract

**Introduction:**

Reimbursement schemes should be regularly updated to maintain a trade-off between costs for the system and access to medicines. The aim of this study was to review reimbursement systems in several European countries in terms of solutions that could have a positive impact on the health technology assessment (HTA) and reimbursement processes in Poland (e.g., increasing patient access to medicines while maintaining payers’ spending).

**Methods:**

Secondary publications and the websites of key institutions responsible for drug policy (the Ministry of Health, HTA agencies, and payers) in selected European countries were searched to find unique solutions that could have a positive impact on drug policies in Poland. The keywords used were “drug reimbursement” and “HTA”. A more specific search was conducted as needed.

**Results:**

The following solutions considered worthy of further consideration:

- central organization of tenders for hospital drugs and conducting price negotiations with regional financial responsibility;
- determining the maximum annual copayment per patient for reimbursed drugs;
- complementary private health insurance that reduces patient copayments;
- creating a separate path for hybrid drugs in the HTA and pricing processes;
- increasing the number of consultations with the market authorization holder, clinical experts, and the public during drug evaluation; and
- not accounting for the costs of lost productivity during HTA due to the discrimination of seniors and children.

**Conclusions:**

Minor changes in the HTA process, such as increasing the role of consultations, as well as major systemic changes (e.g., introducing complementary private insurance, creating a separate path for hybrid drugs, and introducing a maximum annual copayment for reimbursed drugs) could improve patients’ access to drugs. Implementing these solutions requires significant adaptation of the local legal framework.

